# Oro-facial manifestations of 100 leprosy patients

**DOI:** 10.4317/medoral.17718

**Published:** 2012-02-09

**Authors:** Jamileh B. Taheri, Hamed Mortazavi, Mahkameh Moshfeghi, Mahin Bakhshi, Sedigheh Bakhtiari, Saranaz Azari-Marhabi, Somayeh Alirezaei

**Affiliations:** 1 Associate Professor, Department of Oral Medicine, Faculty of Dentistry, Shahid Beheshti University of Medical Sciences, Tehran, Iran; 2 Assistant Professor, Department of Oral Medicine, Faculty of Dentistry , Shahid Beheshti University of Medical Sciences, Tehran, Iran; 3 Assistant Professor, Department of Oral and Maxillofacial Radiology, Faculty of Dentistry, Shahid Beheshti University of Medical Sciences, Tehran, Iran; 4 Resident, Department of Oral Medicine, Faculty of Dentistry, Shahid Beheshti University of Medical Sciences, Tehran, Iran

## Abstract

Objectives: To verify the frequency of oral and facial involvement in diagnosed leprosy patients. Study design: This study was performed on 100 leprosy patients (62 male, 38 female, mean ages 51.86±6.1). After explaining the study design, we studied descriptive information including: patient’s sex, age, job, place of birth, familial history of leprosy, types of disease (lepromatous, borderline and tuberculoid leprosy), ocular and oral lesions, facial involvement and neuropathy. The statistical signification was measured by chi-square test. Results: A total of 46 (23 lepromatous, 15 borderline, and 8 tuberculoid leproy) out of 100 patients with leprosy had oral lesions. Statistical analysis did not show any significant difference in frequency of oral lesions between different types of disease. Facial lesions were presented in 57 (39 lepromatous, 10 borderline, and 8 tuberculoid leprosy) patients. There was a statistical significant difference in frequency of facial manifestations between different types of leprosy. It has to be mentioned that, atrophy of nasal spine, facial nerve involvement, ocular lesions and facial deformity were seen in 15%, 17%, 22% and 44% of leprosy patients, respectively. Conclusion: Examination of leprosy patients should be extended to the oral mucosa because oral mucosa may be a secondary source of M.Leprae transmission and infection.

** Key words:**Leprosy, lepromatous, tuberculoid, oral lesions, facial lesions.

## Introduction

Leprosy is a chronic infectious contagious disease produced by Mycobacterium Leprae (M. Leprae). It mainly affects the skin and peripheral nerves. Involvement of internal organs and mucosa has been also reported (1,2). Leprosy is still a public health problem in many areas of the world; over than 80% of all reported cases are noted in seven countries: Brazil, India, Indonesia, Madagascar, Myanmar, Nepal and Nigeria (3-5).

Clinical presentations of leprosy are related to the immune response against M. Leprae. The first, tuberculoid leprosy (paucibacillary; TT) that is characterized by high immune reaction to the organism, a few cutaneous lesions and little number of bacilli in skin biopsy specimens. The second, lepromatous leprosy (multibacillary; LL), that usually develops in patients with reduced cell-mediated response and negative lepromin skin tests (2,6,7). Within this spectrum there are borderline and less common variations with intermediate lesions. The main clinical features of leprosy are listed as follows: erythematous or hypopigmented macules on the skin with reduced sensation, distinct sensory loss and peripheral nerve involvements, muscle weakness, nerve thickening, positive skin biopsy of acid – fast bacilli and positive skin smear. It is noted that a subject with one or two of these major findings should be considered as a leprosy case (8).

Oral lesions are rare in this disease but when present occur in lepromatous form (6,9). These lesions initially appears as yellowish to red, sessile, firm, papule or nodules that develop ulceration and necrosis. Continuous infection can lead to scarring and tissue destruction (3,6). Unfortunately, in contrast to cutaneous manifestations of leprosy that are well discussed in medical literature, there are few studies around the oral findings in leprosy patients (2,10). Recently, it has been suggested that oral mucosa may be a secondary source of M. Leprae infection and transmission (1).

Also, it is noted that there is an association between the recurrence of leprosy reactional episodes and oral manifestations in leprosy patients (11). For these reasons, oral examinations should be done in these patients.

## Material and Methods

This study was performed at the Shahid Bolandian Health center of Qazvin, Iran. A hundred leprosy patients (62 male, 38 female, mean ages 51.86±6.1) were selected. After explaining the study design, written informed consent was obtained from all participants. Then we studied descriptive information including: patient’s sex, age, job, place of birth, familial history of leprosy, types of disease (lepromatous, borderline and tuberculoid leprosy), ocular and oral lesions, facial deformities and neuropathy. Previous treatment was an exclusion criterion in present report. The data analysis was done using SPSS version 13 for windows and the statistical signification was measured by chi-square test. P-value less than 0.05 (p<0.05) were considered as statistically significant.

## Results

Among 100 leprosy patients, 62 (62%) were male and 38 (38%) were female, with ages ranging from 7 to 87 years. However, most of the patients (77%) were between 30 and 70 years old ( Table 1). More than half of the subjects (61%) were farmer and about 40% of them were born in Gylan province, city of Rudbar, North of Iran.

**Table 1:**
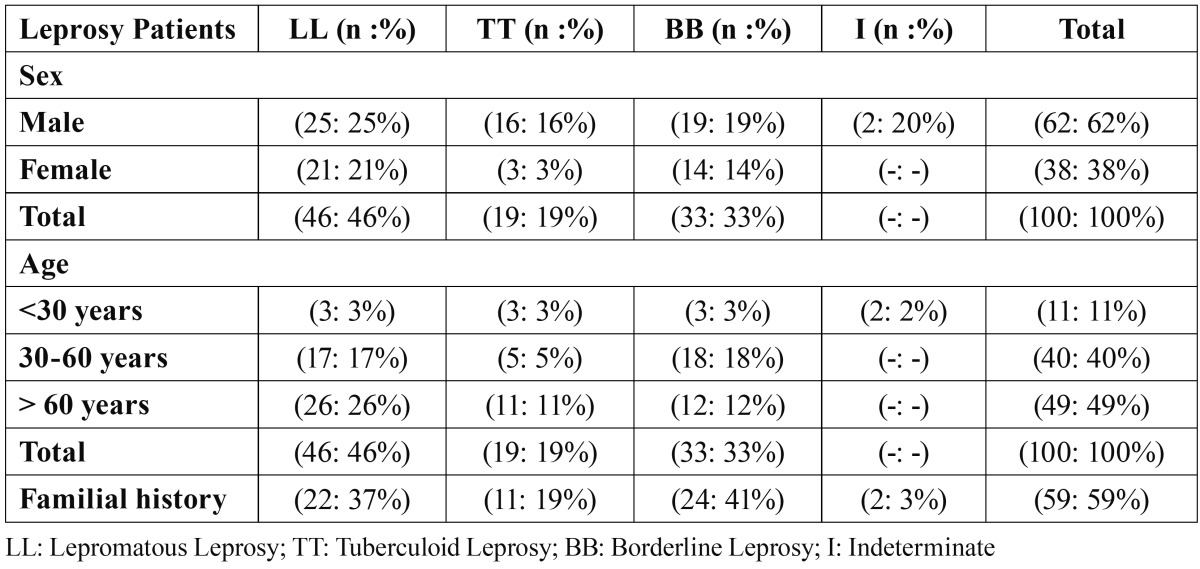
Partakers’ self-assessment of their capacities to undertake a SFE (Likert 1-5 scale).

The different types of disease in this study were: 2 (2%) indeterminate, 33 (33%) border line, 46 (46%) lepromatous and 19 (19%) tuberculoid leprosy.

Familial history of leprosy was observed in 59 (59%) cases ( Table 1). Most of the patients had their leprosy diagnosed 8 months after the first signs and symptoms appeared.

A total of 46 (23 lepromatous, 15 borderline, and 8 tuberculoid leproy) out of 100 patients with leprosy had oral lesions.

Statistical analysis did not show any significant difference in frequency of oral lesions between different types of disease (p=0.390).

In this study, facial lesions were presented in 57 (39 lepromatous, 10 borderline, and 8 tuberculoid leprosy) patients. Obviously, there was a statistical significant difference in frequency of facial manifestations between different types of leprosy (p=0.002). It has to be mentioned that, atrophy of nasal spine, facial nerve involvement, ocular lesions (Lagophthalmous, keratitis, and conjunctivitis) and facial deformity were seen in 15%, 17%, 22% and 44% of leprosy patients, respectively.

Orofacial manifestations of leprosy in different types of disease were summarized in  Table 2.

**Table 2:**
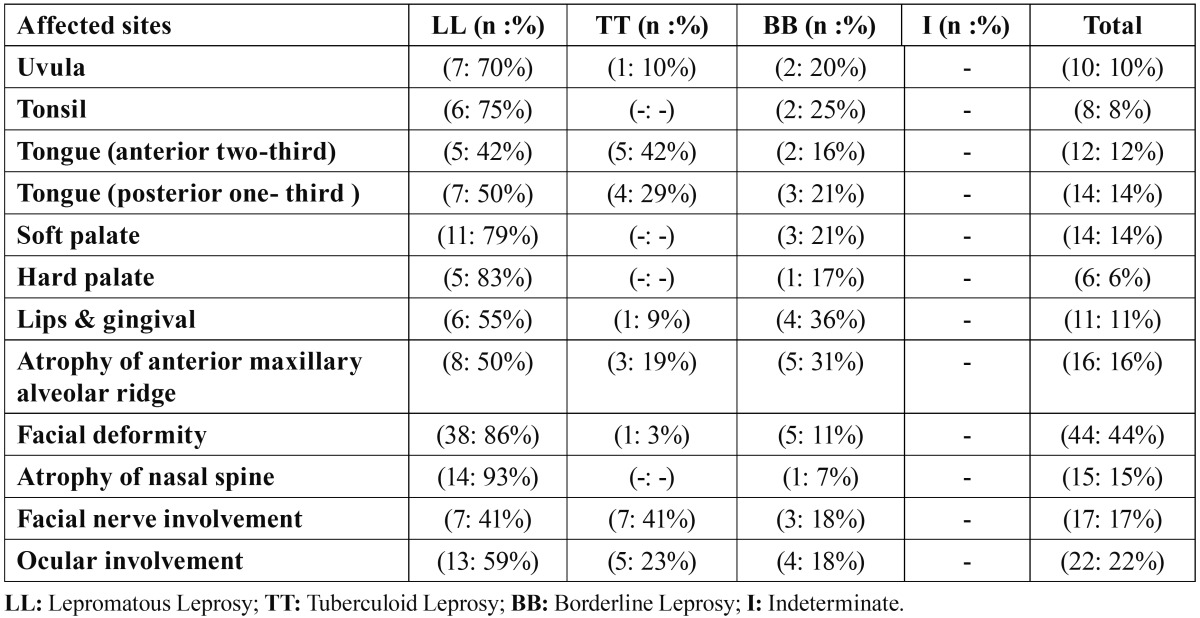
Distribution of Oro-facial lesions in 100 Leprosy patients.

## Discussion

The upper airways are the most important point of entry for bacillus and a main source for bacillary elimination in leprosy (6,12). In recent studies, oral mucosa seems to be the secondary (after nasal mucosa) site of M. Leprae infection and transmission (1,2). Also, involvement of oral mucosa may have a key role in leprosy transmission from adults to children (13). Indeed, it is established that these lesions tended to be more frequent over the first 5 years of the disease (3,10).

For these reasons, evaluation of oral cavity should be routine for leprosy patients (2,6).

In this present study we evaluated 100 leprosy patients, the age of them ranged from 7 to 87 years, with average of 51.86±6.1. The number of male subjects (62: 62%) was higher than females (38:38%).

This finding is in agreement with de Abreu et al.(2) and Boggild et al.(13). In contrast to this result, Souza et al.(10) did not show any difference between men and women in leprosy.

The mean age of our leprosy patients was close to previous studies such as de Abreu et al.(2) and Souza et al.(10). In this study the rate of lepromatous leprosy was higher than other types of disease and it was more common in males than females. These findings are in accordance to Boggild et al.(13) and Toweir et al.(14).

 In our study, familial history of leprosy was reported by 59% of patients which most of them (41%) had borderline leprosy. Also, the number of patients with familial history of lepromatous leprosy was higher than tuberculoid leprosy. This finding may be related to the close contact with carriers of the bacillary (10).

**Figure 1:**
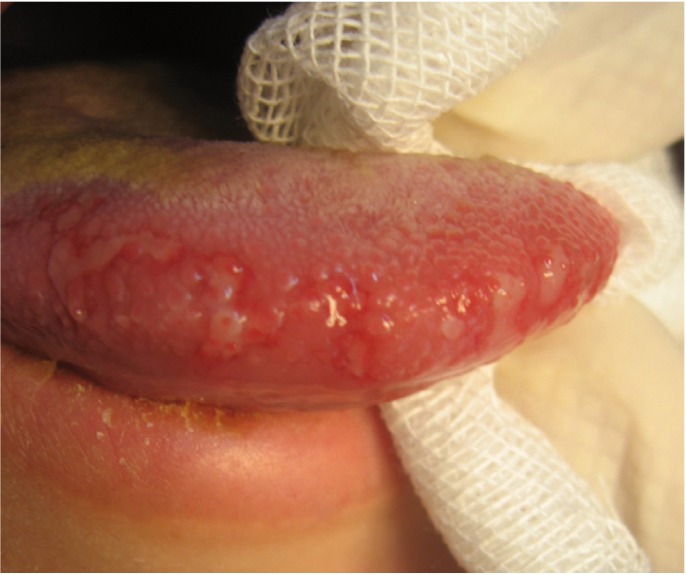
Oral lesion involving the tongue in tuberculoid leprosy.

**Figure 2:**
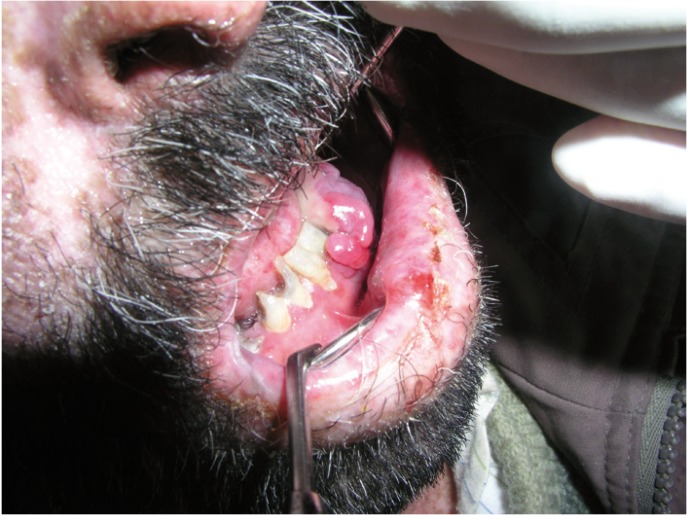
Tumor like lesion in lepromatous leprosy patient.

**Figure 3:**
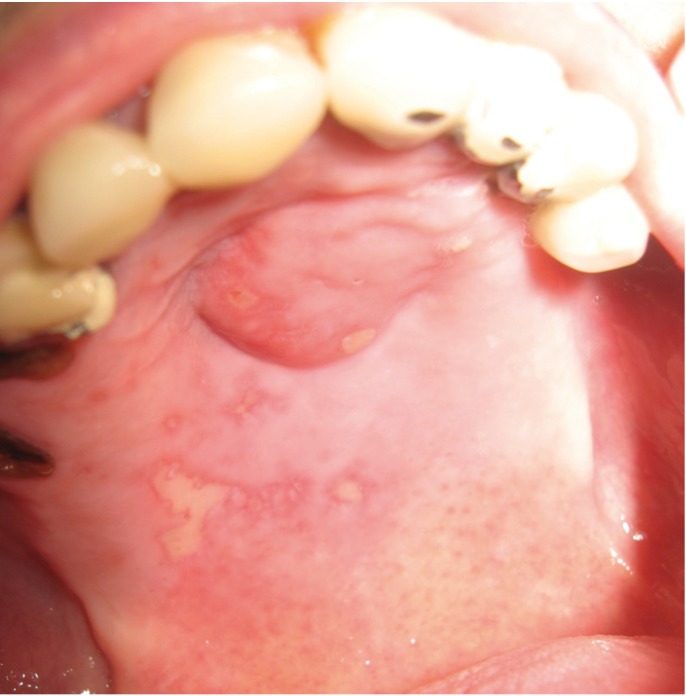
Oral lesion involving the palate in lepromatous leprosy.

According to our results, oral involvement was observed in 46% of all studied cases (Figs. 1,2,3). It was more frequently in patients with lepromatous leprosy. Unfortunately, review of literature showed a discrepancy in frequency rates of oral involvement in leprosy. For example, oral lesions in leprosy patients have been reported from absent up to 57.5% (2,15,16).

On the other hand, Prabhu et al.(17), in an excellent review of 700 leprosy patients showed that the prevalence of oral involvement in leprosy was 11.5% and also these lesions tended to occur in lepromatous leprosy. Some authors such as Motta et al.(6), Soni (18) and Palaskar (19) established that the oral lesions usually appear in the advanced stage of leprosy.

In contrast to this hypothesis, de Abreu et al.(2), Brasil et al.(20) and Sharma et al.(21) demonstrated that oral lesions may occur in less advanced stages of the disease and also in these patients oral cavity involvement remains clinically hidden (absence of visible lesions) and may only detected histopatholosically. This can be one of the main explanations for different rates of oral involvement in leprosy seen in literature (2).

It is suggested that oral involvement in leprosy occurs by hematogenous or lymphatic dissemination of M. Leprae (6,22).

On the other hand, Bucci et al.(23) and Girdhar et al.(24) pointed out that nasal lesions may be precursors of oral lesions.

In our study, of 46 patients with oral lesions, 8 had tuberculoid leprosy. Oral involvement in this type of disease is rare and it may be related to the intense response of the host’s immune system and little number of organisms in this type of disease (8). This finding is in disagreement with de Abreu et al. (25).

de Abreu et al.(25), demonstrated that paucibacillary leprosy patients do not exhibit clinical or subclinical, involvement in the oral cavity. The distribution of oral lesions in our study are ranked as follows: soft palate, tongue (posterior one – third), tongue (anterior two –third), lips, uvula, tonsil, hard palate (Table 2). According to WHO, the most affected sites of oral cavity in leprosy patients are: hard palate, soft palate, labial maxillary gingival and buccal mucosa (26). In this present report, involvement of tonsil, soft palate and hard palate was not observed in patients with tuberculoid leprosy.

Similar to our finding, Motta et al.(6), Brasil et al.(20) and Reichart (27) showed that the soft palate was affected most frequently in almost all cases of leprosy.

In this present study facial involvement was observed in 39 (39 %) leprosy patients. This rate was 28% in Prabhu’s study (17). The frequency and distribution of facial involvements have been summarized in table 2.

According to our findings, atrophy of nasal spine was not found in patients with tuberculoid leprosy. Also, ocular involvement was higher in lepromatous leprosy than other types of disease. Furthermore, the rate of facial nerve involvement was equal in lepromatous and tuberculoid leprosy.

In conclusion, examination of leprosy patients should be extended to the oral mucosa because oral mucosa may be a secondary source of M.leprae transmission and infection.
